# Knowledge, attitude and practice of animal producers towards antimicrobial use and antimicrobial resistance in Oromia zone, north eastern Ethiopia

**DOI:** 10.1371/journal.pone.0251596

**Published:** 2021-05-12

**Authors:** Daniel Teshome Gebeyehu, Demisew Bekele, Belay Mulate, Getachew Gugsa, Tarekegn Tintagu

**Affiliations:** 1 Department of Veterinary Medicine, School of Veterinary Medicine, Wollo University, Dessie, Ethiopia; 2 Department of Veterinary Laboratory Technology, School of Veterinary Medicine, Wollo University, Dessie, Ethiopia; University of Lincoln, UNITED KINGDOM

## Abstract

Antimicrobial resistance is the failure of antimicrobial’s effect against the growth and multiplication of microorganisms. Imprudent and over antimicrobial use (AMU) aggravates antimicrobial resistance (AMR). Antimicrobials are massively used in animal production as compared with AMU in human health sectors. This research was done with the objective of assessing the knowledge, attitude, and practice (KAP) status of animal producers towards AMU and AMR. A Cross-sectional study design and questionnaire were conducted and both qualitative and quantitative data analyses were used. The logistic regression was used to test the effect of each predictor variable on the knowledge, attitude, and practice of the participants. Out of 571 animal producers, the majority (80.2%) of them were not knowledgeable and 85.3% of the animal producers had a negative attitude towards the AMU and AMR. Likewise, the practice of 78.5% of the animal producers were practice improperly towards AMU and AMR. All the questions that were designed to assess the KAP of the animal producers were significantly associated (P<0.05) with each respective category of KAP. The educational status of animal producers was negatively correlated (OR = 0.38) with all their knowledge, attitude, and practice of AMU and AMR, but sex has a positive correlation (OR = 2.89) with both the knowledge and practice of animal producers. In conclusion, the animal producers in the Oromia zone had unsatisfactory knowledge regarding AMU and AMR. The animal producer’s attitude and their practices were negative and improper respectively. As a result, consecutive awareness creation on both AMU and AMR is recommended and integrated AMU governance in animal production is recommended to be applied.

## Introduction

According to the definition of the American Veterinary Medical Association [1], “antimicrobials are agents that kill microorganisms or suppress their multiplication or growth”. The failure of antimicrobial’s effect against the growth and multiplication of microorganisms is called antimicrobial resistance (AMR). AMR happens when “microorganisms (such as bacteria, fungi, viruses, and parasites) change when they are exposed to antimicrobials (such as antibiotics, antifungals, antivirals, antimalarial, and anthelmintic)” [2].

Even if different factors aggravates AMR, over-use, and misuse of antimicrobials play a major role. Indeed, AMR emergence and diffusion correspond to a selective process allowing microbial populations to adapt to their environment. Therefore, AMR is inextricably tied to all forms of antimicrobial use (AMU) and will be favored when this use is sub-optimal or widespread. In particular, avoidable practices that are recognized as key contributors to AMR are AMU in animal production for growth promotion, prophylaxis, and metaphylaxis; AMU without professional oversight; and or AMU after poor diagnostic techniques [3]. As explained in FAO’s 2016–2020 AMR action plan, every sector and actor regardless of their economic status and their geographic locations are affected by AMR [4].

Antimicrobial-resistant pathogens can circulate in populations of humans and animals, through food, water, and the environment [5]. Transmission of AMR is facilitated by trade, travel, and both human and animal movement. Resistant microbes can be found in food animals and food products destined for consumption by humans [6]. Since the need for animal origin protein is increasing, AMU as food animal growth promotion is greater than 4 times the use of antimicrobials in humans [5]. Farmers, especially animal producers supply many antimicrobials by mixing them with feed and/or water to their animals. As a result, the prevalence of AMR in food animals was higher than in non-food animals (Canines, equines, and feline species) [7]. Using antimicrobials in animal production results in AMR development and drug residue in foods of animal origin [8]. Infections caused by antimicrobial-resistant pathogens are difficult to treat. Due to AMR, patients are exposed to longer periods of stay in the hospital, non-affordable and toxic last-resort drugs, and unsuccessful surgical operations [9]. As a result, the health risks of antimicrobial-resistant pathogens are more severe than non-resistant ones.

As recommended by the American Veterinary Medical Association [1], “judicious use of antimicrobials in food-producing animals” should target the use of antimicrobials when it is mandatory for the treatment, prevention, and control of diseases with a confirmed diagnosis. In the opposite of this recommendation, antimicrobials are carelessly used in the animal-origin food chain in different corners of the world. To limit the contribution of animal production to the global health threat of AMR, multi-sectoral and integrated awareness creation to animal producers is needed and should be applied across a diversity of countries, showing a diversity of status of economic development of their livestock sectors [6]. For creating awareness to animal producers and formulating appropriate legislation towards the use of AMU and AMR, assessing the knowledge, attitude, and practice of animal producers is a foundation. The study area (Oromia zone) was chosen due to the common practice of illegal antimicrobial trades and poor quality drug circulation in it.

Therefore, the objective of this study was to assess the status of the livestock animal producers’ knowledge, attitude, and practice towards AMU and AMR in the Oromia zone.

## Methodology

### Study area and population

The study was conducted in the Oromia zone, which has an area of 3,470 km2 with a population of 457, 278 [10]. According to the national statistics agency 2016/17 agricultural sample survey, the Oromia zone has 288, 941 Cattle, 279, 456 Poultry, 241, 399 Goats, 116, 388 Sheep, 48, 856 Donkeys, 17, 751 Camels, 161 Horses, and 161 Mules. The climatic condition of the study area ranges from hot to warm weather. Since the Oromia zone is very near to the port of Djibouti, it is expected to be the entrance site of antimicrobials from abroad to Ethiopia. As a result, the zone was chosen due to expectations of illegal antimicrobial trade and massive antimicrobial use for animal production, which can aggravate the AMR formation. Oromia zone has seven districts: namely Artuma Fursi, Bati, Dawa Chefe, Dawa Harewa, Jile Timuga, Kemise, and Senbete. From these districts, four districts (Bati, Dewa Chefa, Jule Timuga, and Kemissie) were randomly selected by lottery method for sample collection. The study population was animal producers who were aged > 18 years, present in the livestock market at the time of study, and producing food animals (Cattle, Sheep, Goat, and Poultry). Other animals in the study area are not considered food animals due to religious and cultural taboos.

### Data collection

Structured questionnaire interviews were conducted to assess the knowledge, attitude, and practice (KAP) of AMU and AMR of animal producers in the Oromia zone. In each selected district of this zone, the largest open livestock market was chosen and the animal producers in these markets were invited for interview. All animal producers regardless of their educational status and sex were included as information sources for the study.

The questionnaire had four sections and contained close-ended questions (S2 File). The first section was about the respondents’ socio-demographic characteristics such as age, sex, educational level, districts, animal type reared by respondents, and respondents’ residence. These six variables have their own categories. The second section was focused on the knowledge of animal producers’ AMU and its externalities in terms of AMR. This section has six categorical variables that enables us to assess the knowledge of the animal producers. The third section of the questionnaire was designed for assessing the respondent’s attitude about AMU and AMR. The fourth section was targeted at the practice of the animal producers about their AMU and the contribution of AMU in animal production to AMR development. The responses of the animal producers about their AMU and AMR KAP were categorized into different categories under each type of question.

### Study design and sample size

A cross-sectional study was conducted on the animal producers’ KAP towards AMU and AMR in selected districts of the Oromia zone. The sample size for assessing animal producers’ KAP towards AMU and AMR was calculated based on the suggestions of Bartlett *et al*. [11]. Bartlett and his research team suggested that for every type of cross-sectional survey the following formula is more appropriate than others.

$$\frac{n=p\;(100-p)\;z^{2}}{e^{2}}$$

Where n = is the required sample size

p = is the percentage occurrence of a state or condition

z = is the value corresponding to level of confidence required

e = is the percentage maximum error required

Since there was no study done about the KAP of animal producers towards AMR and AMU in the study area, 50% for p-value, 95% (1.96) for z-value, and 5% for e-value were taken. As a result, the sample size was calculated as follows.

$$\begin{array}{c} \frac{n=50(100-50)1.96^{2}}{5^{2}}\\ =384\;\mathrm{samples}\;\mathrm{for}\;\mathrm{each}\;\mathrm{assessment} \end{array}$$

The researchers collected a higher number of samples than the calculated minimum number of samples (384). The total sample size from Bati, Dewa Chefa, Jule Timuga, and Kemissie were 182, 135, 120, and 134 respectively. As a result, the total sample size for this study was 571.

### Ethical considerations

The ethics concerned with this research activity were evaluated by the institutional review board (IBR) of Wollo University. For conducting this research the researchers were requested a support letter from Wollo University, school of veterinary medicine and received a letter with the reference number WU/SVM/212/10. Participants of the study were given all the information regarding the study and signed the informed consent form before they were recruited into the study.

### Data analysis

In this study, both descriptive and inferential analyses were used. After the required number of samples were collected, it was administered in Microsoft Excel 2013. The dataset was composed of 24 categorical variables (six variables each part of demographic, knowledge, attitude, and practice). Based on Likert’s scale, dependent binary variables were produced for each category of KAP from each respective variable. The participants who correctly answer all knowledge-oriented questions were categorized as knowledgeable and those who fails to answer one or more were grouped as not knowledgeable. Likewise, all respondents whose attitude was towards the mitigation of AMR development in all attitude-based questions were grouped into a positive attitude and the respondents who had attitudes that promote AMR development were categorized into a negative attitude. In the same way, all the participants who were practiced towards the reduction of AMR were grouped into good practice and those who practice in the opposite were categorized into bad/improper practice. Each participant was assigned to the newly formed KAP categories by using a pivot table. The purpose of producing these three dependent variables was for assessing the effect of each predictor variable on KAP using logistic regression. Since 3 binary variables (KAP variables) were formed in addition to the 24 categorical variables, the dataset totally contains 27 variables S1 File.

The binary logistic regression was used to see which explanatory variables are predictive for the result, knowledgeable or not knowledgeable; good practice or improper practice; and positive attitude or negative attitude. Our investigation of the animal producers’ knowledge, attitude, and practice on AMU and AMR follows three steps. The first step was assessing the relationship between potential predictor variables with the animal producers’ KAP one by one. Secondly, we adjust the relationship for the potential confounding effects. Finally, we consider the possibility of interaction effects among the variables.

After descriptive investigations using crosstabs, the association between KAP categories (knowledge, attitude, and practice) and each predictive variable was conducted. Likelihood ratio test (LR test), and probability values from SPSS software version 26 were used to see the association between dependent variables (knowledge, attitude, and practice) and predictive variables. The effect level of demographic variables on knowledge, attitude, and practice was shown by the odds ratio. Wald’s chi-square test and probability value were used to see the association between demographic variables with KAP.

## Results

### Demographic characteristics of the animal producers

Half (50.3%) of the participants were semi-urban residents, and 47.7% of the respondents were in the age group of 31–40 years. There was a higher number (56.9%) of male participants than females (43.1%). In the study area, the majority (34.5%) of animal producers had reared goats and a small number (6.5%) of the participants were reared sheep while 22.4% of the respondents were reared all food animals. Food animals in the study area are ruminants (cattle, sheep, and goats), camels, and poultry only. Due to cultural and religious issues, other species of animals are not allowed to eat in the study area. The highest number (32.9%) of the participants were not educated (illiterate) and others were attended from primary to tertiary education levels (Table 1). The details of the demographic characteristics of the respondents are described in Table 1.

**Table 1:** Animal producers’ demographic characteristics.

Characteristics	Categories	Number	Percent (n = 571)
Age	18–30	160	28.0
	31–40	238	41.7
	>40	173	30.3
Sex	Male	325	56.9
	Female	246	43.1
Educational level	Illiterate	188	32.9
	Primary school	160	28.0
	Secondary school	123	21.5
	Tertiary education	100	17.5
Residence	Urban	120	21.0
	Semi Urban	287	50.3
	Rural	164	28.7
District	Bati	182	31.9
	Dewa Chefa	135	23.6
	Jule Timuga	120	21.0
	Kemissie	134	23.5
Animal type they reared	Cattle	139	24.3
	Sheep	37	6.5
	Goat	197	34.5
	Poultry	70	12.3
	All animal types	128	22.4

**Fig 1:**
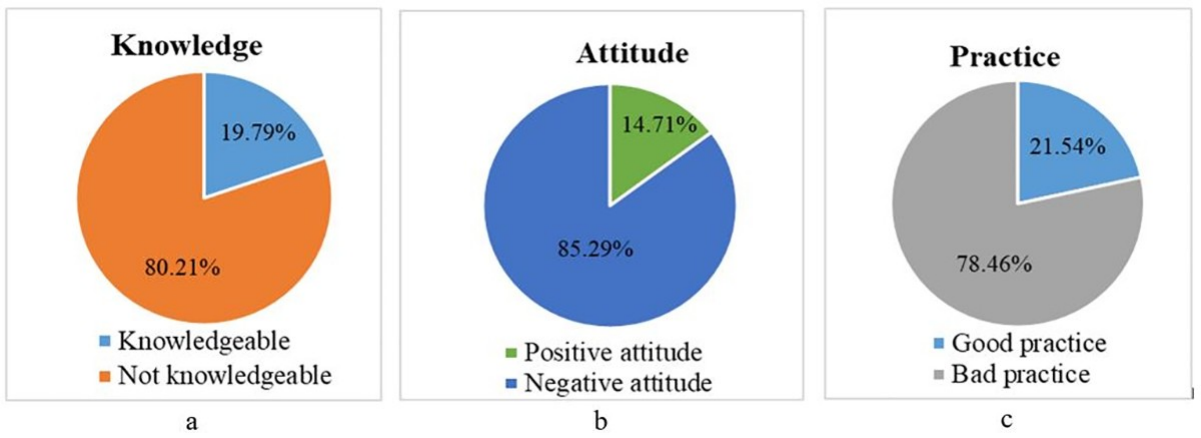
Knowledge, attitude, and practice of animal producers towards AMU and AMR.

The majority (80.2%) of the participants were not knowledgeable about antimicrobial use in animal productions and its negative consequence (AMR) (Fig 1A). A large number (85.3%) of the animal producers had a negative attitude towards the AMU and AMR (Fig 1B) while 78.5% of the respondents had improper practice on the AMU and aggravates AMR (Fig 1C).

### The knowledge of animal producers towards AMU and AMR

The majority (55.9%) of the animal producers didn’t know about rational AMU and the prevention of AMR formation. Thirty-four percent of the animal producers were not aware of AMU in animal production can able to aggravate AMR. Forty-one percent of the participants know that their imprudent use of antimicrobials in animal production can have negative externality to society in the form of AMR. The details of the animal producers’ knowledge and the statistical associations are presented in Table 2.

**Table 2:** The knowledge of animal producers towards AMU and AMR in animal production.

Statements/Questions	Responses	Percent (n = 571)	LR test (X^2^)	P value
Do you know or heard of AMU and AMR?	Yes	44.1	221.46	0.0001
	No	55.9		
Can zoonotic diseases causing agents to develop AMR in animals?	Yes	43.1	228.72	0.0001
	No	28.7		
	I don’t know	28.2		
Do you know using animal-origin food products before the end of the withdrawal period can promote AMR development in humans?	Yes	39.8	253.43	0.0001
	No	25.7		
	I don’t know	34.5		
Can the use of antimicrobials in animal production boost the rate of AMR development?	Yes	38.0	267.66	0.0001
	No	27.7		
	I don’t know	34.3		
Can you reduce AMR development by avoiding the over-use of antimicrobials in animal production?	Yes	35.0	294.25	0.0001
	No	27.1		
	I don’t know	37.8		
Can your imprudent use of antimicrobials affect the health of others in the form of AMR?	Yes	41.2	242.68	0.0001
	No	28.5		
	I don’t know	30.3		

n = total number of samples; LR = likelihood ratio; X^2^ = Chi-square value; the higher likelihood ratio test score indicates the higher influence of the animal producers’ response to be knowledgeable or not knowledgeable towards AMU and AMR.

As showed in Table 2, all the six AMU and AMR-related questions were significantly associated (p<0.05) with the cumulative knowledge of the respondents.

### The attitude of animal producers towards AMU and AMR

As indicated in Table 3, the Likert scale was used to test the attitude of the respondent about AMU and AMR with five modalities (strongly agree, agree, neutral, disagree and I don’t know). There was one extra disagreement modality in the questionnaire (strongly disagree), but it was not chosen by all the participants and it is not included in Table 3. All the six attitude-related questions were statistically significant (p<0.05) with the cumulative attitude of the respondents. In addition to the respondents’ level of agreement, the statistical association of each variable with the cumulative attitude of the participants is presented in Table 3.

**Table 3:** The attitude of animal producers towards AMU and AMR in animal production.

Questions	Responses	Percent (n = 571)	LR test (X^2^)	P value
Is professional advice before using antimicrobials recommended?	Strongly agree	39.2	149.17	0.0001
	Agree	13.5		
	Neutral	21.0		
	Disagree	18.7		
	I don’t know	7.5		
Can imprudent AMU result in irreversible loss of drug effectiveness?	Strongly agree	11.4	346.60	0.0001
	Agree	12.1		
	Neutral	12.8		
	Disagree	28.9		
	I don’t know	34.9		
Can using antimicrobial alternatives like biosecurity, good hygienic practice and vaccination can reduce AMR development?	Strongly agree	11.6	268.78	0.0001
	Agree	17.0		
	Neutral	8.8		
	Disagree	44.1		
	I don’t know	18.6		
Do you think using antimicrobials for the purpose of animal production is abusing antimicrobials?	Strongly agree	6.8	379.36	0.0001
	Agree	11.2		
	Neutral	16.8		
	Disagree	23.1		
	I don’t know	42.0		
Can AMU regulations will be a solution for the irrational use of antimicrobials in animal production?	Strongly agree	12.3	319.35	0.0001
	Agree	9.6		
	Neutral	13.0		
	Disagree	35.9		
	I don’t know	29.2		
Can public awareness creation reduce the development of AMR?	Strongly agree	46.6	113.34	0.0001
	Agree	31.3		
	Neutral	12.4		
	Disagree	4.9		
	I don’t know	4.7		

n = total number of samples; LR = likelihood ratio; X^2^ = Chi-square value; the higher likelihood ratio test score indicates the higher influence of the animal producers’ response to have positive or negative attitude towards AMU and AMR.

### The practice of animal producers towards AMU and AMR

The animal producers’ AMU and AMR practices were tested using six practice-related questions (Table 4). The majority (53.9%) of the animal producers had used prescriptions for buying antimicrobials while the remaining (46.1%) were purchasing antimicrobials without prescription. As described in Table 4, 39.4% of the participants were administered drugs by themselves to their animals. The majority (41.5%) of the participants used antimicrobials for treatment purposes. All six questions were significantly associated (p<0.05) with the cumulative practice of animal producers (Table 4).

**Table 4:** The practice of animal producers towards AMR and AMU in animal production.

Questions	Responses	Percent (n = 571)	LR test (X^2^)	P value
What did you do when your animals got sick?	Self-treat	28.0	167.79	0.0001
	Take to vet clinic	44.3		
	Consult veterinarian	27.7		
Who administer antimicrobials for your animals?	Self	39.4	188.91	0.0001
	Veterinarian	52.5		
	Local traditional healer	8.1		
Did you refer to guidelines while you administer antimicrobials for your animals?	No	43.3	164.83	0.0001
	Yes	56.7		
Did you get a prescription from veterinarians before you buy drugs?	No	46.1	180.61	0.0001
	Yes	53.9		
For what purpose did you use antimicrobials most?	Treatment	41.5	266.82	0.0001
	Control (Metaphylaxis)	14.9		
	Prevention (Prophylaxis)	24.2		
	Increase production	19.4		
From where did you get antimicrobials for your animals?	Local dispensers	19.8	68.10	0.0001
	Veterinary clinic	34.9		
	Veterinary Pharmacy	45.4		

n = total number of samples; LR = likelihood ratio; X^2^ = Chi-square value; the higher likelihood ratio test score indicates the higher influence of the animal producers’ response to having improper or good practice towards AMU and AMR.

### The association of demographic variables to the KAP of farmers

The residence, district, age, sex, educational level of the animal owners, and the animal type they reared were the demographic categorical variables. The categories under each categorical variable were taken as the dummy variables and one of them was considered as a reference dummy variable. All the dummy variables of the variable “educational level” were significantly associated (p<0.05) with all the knowledge, attitude, and practice of the participants. The educational level of animal producers was negatively correlated (OR < 1) with their KAP towards AMU and AMR (Table 5). That means, the animal producers with the lower academic level were not knowledgeable about AMU and AMR in animal production. Only the variable “sex” was positively correlated with both the knowledge (OR = 3.49) and the practice (OR = 2.89) of animal producers. This means male animal producers were knowledgeable towards AMU and AMR as compared with female. Except for the sex and the educational level of the participants, all other demographic variables (residence, district, age, and animal type) were not associated with the KAP of animal producers. The details of the statistical associations (Wald’s chi-square test, P-value, Odds ratio, and the confidence interval for the odds ratio) are presented in Table 5.

**Table 5:** The association of demographic dummy variables with the animal producers’ KAP towards AMU and AMR.

Categorical predictor dummy variables	Knowledge			Attitude			Practice		
	Wald’s X^2^- test	P-value	OR(95% CI)	Wald’s X^2^- test	P-value	OR(95% CI)	Wald’s X^2^- test	P-value	OR(95% CI)
**Residence**	0.71	0.70		4.37	0.11		0.79	0.67	
Semi Urban vs. Urban	0.55	0.46	1.25(0.7–2.23)	4.32	0.04	2.05(1.04–4.03)	0.18	0.68	0.89(0.50–1.56)
Rural vs. Urban	0.005	0.95	1.02(0.62–1.66)	2.17	0.14	1.57(0.86–2.84)	0.79	0.38	0.81(0.51–1.29)
**District**	3.08	0.38		3.33	0.34		4.78	0.19	
Dewa Chefa vs. Bati	1.50	0.22	1.43(0.81–2.55)	0.07	0.79	1.09(0.59–1.97)	3.71	0.05	1.73(0.99–3.02)
Jule Timuga vs. Bati	0.02	0.89	1.05(0.55–1.98)	1.58	0.21	0.64(0.31–1.29)	0.08	0.77	1.09(0.59–2.05)
Kemissie vs. Bati	1.89	0.17	1.55(0.83–2.89)	0.78	0.38	0.73(0.36–1.48)	1.13	0.29	1.40(0.75–2.61)
**Age**	0.50	0.78		1.57	0.46		1.97	0.37	
31–40 vs.18-30	0.13	0.72	0.91(0.52–1.57)	1.48	0.22	1.46(0.79–2.67)	0.05	0.83	1.06(0.62–1.82)
>40 vs. 18–30	0.113	0.74	1.09(0.67–1.77)	0.21	0.65	1.14(0.64–2.04)	1.62	0.20	1.37(0.85–2.21)
**Sex**									
Female vs. Male	25.32	*0*.*001*	3.49(2.14–5.68)	1.53	0.22	1.35(0.84–2.18)	21.19	*0*.*001*	2.89(1.84–4.53)
**Educational level**	38.92	*0*.*001*		12.08	*0*.*01*		17.31	*0*.*001*	
Primary vs. Illiterate	32.78	*0*.*001*	0.18(0.1–0.32)	8.42	*0*.*001*	0.39(0.21-.74)	15.44	*0*.*001*	0.32(0.18–0.57)
Secondary vs. Illiterate	21.26	*0*.*001*	0.26(0.14–0.46)	9.07	*0*.*001*	0.36(0.19–0.70)	9.63	*0*.*001*	0.41(0.23–0.72)
Tertiary vs. Illiterate	16.05	*0*.*001*	0.29(0.16–0.53)	5.12	*0*.*02*	0.46(0.23–0.90)	7.47	*0*.*01*	0.43(0.24–0.79)
**Animal type**	6.29	0.18			8.13	0.09		2.66	0.62	
Sheep vs. Cattle	0.15	0.70	1.14(0.59–2.20	0.56	0.46	1.29(0.66–2.55)	0.05	0.83	0.94(0.53–1.66)
Goat vs. Cattle	4.11	0.04	2.43(1.03–5.72)	3.81	0.05	2.42(1–5.87)	2.01	0.16	1.77(0.80–3.89)
Poultry vs. Cattle	2.75	0.12	1.65(0.91–2.98)	0.51	0.47	0.78(0.39–1.54)	0.09	0.77	1.08(0.64–1.82)
All animals vs. Cattle	1.97	0.16	1.70(0.81–3.57)	0.98	0.32	1.49(0.68–3.28)	0.00	0.99	0.00(0.00-)

OR = odds ratio; vs. = versus; CI = confidence interval; P = probability; X^2^ = chi-square score

## Discussion

### The knowledge of animal producers towards AMU and AMR

Out of the total participants (571) of this study, the majority (80.21%) of the animal producers were not knowledgeable about the AMU and AMR in animal production. Comparable with this finding, 70% of the livestock keepers in Ethiopia [12], 90% of the animal producers in Bingol, Turkey [13], 61.2% of layers and pig farmers in Thailand [14], 80% of animal producers in central Ethiopia [15], and 92.5% of livestock and aquaculture owners in Vietnam [16] were not aware of the proper AMU in animal production and had a poor understanding about the AMR formation.

In the present study, 55.9% of the respondents didn’t have prior knowledge about antibiotic resistance. In the opposite of this finding, a high proportion (77%) of the livestock producers in Vietnam [16], and 35.5% of animal producers in central Ethiopia [15] know about AMU, AMR, and its health risks. Thirty-four and a half percent (34.5%) of the animal producers in the present study didn’t know the relationship between AMU and AMR. A higher proportion (80.4%) than the present study, the livestock producers in Vietnam [16] didn’t know the relationship between AMU and AMR. In contrast to our finding, 35.5% of the animal producers in central Ethiopia [15] know about AMU and AMR. This difference could be a result of different AMU and AMR awareness levels of the livestock producers in different localities. In the present study, 41.2% of the livestock producers know that their imprudent AMU in animal productions has negative externality for the community in the form of AMR. Alike this finding, the study conducted in Vietnam [16] revealed that a large proportion (92%) of the livestock producers understood that their irrational AMU in animal production could aggravate the human and environmental health crises. Similarly, the study on the global AMU situation [17] confirmed that imprudent AMU in animal production results in rapid AMR development. As indicated by the same study [17], multidrug-resistant bacteria were increasingly formed due to imprudent AMU in animal production.

A large proportion (39.8%) of the participants in the present study had full information about the withdrawal period of antimicrobials from animal products. Larger than our finding, 97% of animal producers in Vietnam [16] were knowledgeable about the withdrawal period and its relationship with AMR formation. On the contrary, 70% of the animal producers in Ethiopia [12] didn’t have information about the withdrawal periods of antimicrobials from animal products. Thirty-eight percent (38%) of the livestock producers who participated in the present study know that AMU in animal production can boost the development of AMR. The finding of the present study is in agreement with the studies conducted in different parts of the world [17–19] and the tripartite organizations [20]. For this difference the educational level of the participants, exposure of the animal producers to AMU and AMR training, and the AMU and AMR rules and regulations of the research areas.

The animal producers who were not knowledgeable about AMU and AMR had improper AMU and AMR practices in animal production. In the opposite of our finding, the study conducted in Vietnam [16] revealed that 69% of the livestock producers had good knowledge towards AMU and AMR, but this level of knowledge was not translated to practice. The difference in the knowledge, attitude, and practice of the animal producers in different corners of the world is depending on: type of operation; disease dynamics; economic factors; veterinarian consultation; producer’s experience, and awareness about AMU and AMR [18, 19, 21–24].

### The attitude of animal producers towards AMU and AMR

In our finding large number (85.29%) of farmers had a negative attitude (have an attitude of increasing AMR) regarding the use of antimicrobials and AMR in animal production. Lesser (51%) than the present study, the layer and pig farmers in Thailand [14] had a negative attitude regarding prudent AMU and the practices in reducing AMR formation. In the present study, 39.2% of the animal producers strongly agreed with the significance of having professional advice before using antimicrobials in animal production. Larger (74%) than our finding, farmers in African countries [18], and 95% of livestock producers in Vietnam [16] were agreed that AMU in animal production should be done in consultation with veterinarians. Those animal producers who believe in the importance of using professional advice in the reduction of AMR formation and transmission are those who have a positive attitude towards AMU and AMR.

In the present study, 28.9% of the animal producers disagreed that their irrational AMU in animal production could affect the health of others in the form of AMR. Unlike our finding, 63.7% of the dairy farmers in Swiss [25] and 95% of livestock farmers in Vietnam [16] agreed that imprudent AMU in animal production poses a potential risk to public health. A larger proportion (60%) than our finding, the turkey and rabbit farmers in Italy [26] were negatively perceived that AMR occurs when antimicrobials are used in humans only. As described by [23, 27], the prudent antimicrobial use in animal production and proper formulation and enforcement of AMU legislations in different sectors are the best tools for reducing both the human and animal health crisis. In the current study, 35.9% of the animal producers disagreed with the importance of AMU governance for the reduction of AMR development. Comparable to this, 34.2% of the animal producers in Vietnam [16] and 28.6% of the dairy farmers in Swiss [25] didn’t agree with the contribution of AMU governance in the reduction of AMR formation. On the contrary, 57.5% of the livestock farmers in Vietnam [16] agreed that AMU governance in animal production is a crucial application to mitigate AMR development.

In the present study, a large proportion (44.1%) of the animal producers disagreed with the contribution of alternative AMU in animal production (biosecurity, vaccination, and good husbandry and veterinary practices) in the reduction of AMR formation. On the contrary, 96% of the livestock producers in Vietnam [16] and 82% of the livestock keepers in Ethiopia [12] agreed that the alternative to AMU like vaccination could be important for reducing AMR formation. In addition, 47% of turkey farmers and 78% of rabbit farmers in Legnaro, Italy [26] perceived that they can reduce imprudent AMU by applying genetic improvement. Moreover, the Tennessee beef producers confirmed that vaccination is the most important AMU alternative in the beef production sector [21]. On the other hand, 56.7% of the animal producers in central Ethiopia [15] indicated that AMU alternatives are equally important to AMU.

In our study, 23.1% of the farmers didn’t believe that using antimicrobials in animal production is abusing antimicrobials. On the contrary, a large proportion (69.4%) of the livestock farmers in Vietnam [16] believed that AMU in animal production is abusing antimicrobials. As indicated in [17] irrational AMU in animal production can affect all the animal, human, and environmental health systems. The present study revealed that 31.3% and 46.6% of the livestock producers were respectively agreed and strongly agreed that community awareness is a good way to reduce AMU and AMR in animal production. Comparably, the study conducted on Tennessee’s beef farmers showed that community awareness about AMU is a nice tool for reducing AMR [21].

### The practice of animal producers towards AMU and AMR

The AMU and AMR practices of the animal producers in this study were improper (the practices of animal producers in using antimicrobials are towards aggravating AMR) (78.46%). Comparable with this finding, 72.3% of the livestock farmers had improper practices in the study conducted in Ethiopia [12]. Based on the global AMU trends [24], the Asian AMU in animal production will cover 68% of the world’s AMU by 2030. Similarly, 93.8% of the global AMU for fish production is covered by Asia and only China covers 57.9% of AMU use consumption [28]. These figures and our findings indicated that AMU in animal production is increasing globally and AMR formation is accelerating at an alarming rate. On the contrary, the study conducted on “the unintended consequence of AMU restriction” [29], justified that AMU restriction can promote microbial infection and reduction in job creation.

In the present study, the majority (53.9%) of the animal producers had a trend of using prescriptions before purchasing antimicrobials. Comparable to our finding, 48% of turkey and rabbit farmers in Italy [26], 48% of the animal producers in Turkey [13], and the same proportion (48%) of the livestock producers in Vietnam [16] were seeking the advice of a veterinarian before using antimicrobials for any purpose of animal production. Somehow greater than our finding, 64% of the animal producers in Turkey [13] took advice from their colleagues and purchase antimicrobials without prescription. As indicated by different studies [21, 23], accessing antimicrobials without prescription and fragmented governance of AMU in animal production are the main drivers of AMR formation.

In the current study, a large proportion (41.5%) of the animal producers have used antimicrobials for treatment purposes. Likewise, a high percentage (69%) of the livestock farmers in Vietnam [16], 38% of the livestock farmers in African countries [18], and a lesser percentage (28.6%) of farmers in Swiss [26] confirmed that they mainly use antimicrobials for treatment purpose. In our finding, a large number (45.4%) of animal producers have purchased antimicrobials from veterinary pharmacies while 34.9% and 19.8% get antimicrobials from veterinary clinics and local drug dispensers respectively. Alike this finding, the source of antimicrobials for the majority (89%) of livestock producers in Vietnam [16] and 83% of livestock farmers in different African countries [18] get antibiotics from agro-vet pharmacies. In the present study, the majority (52.5%) of the animal producers’ animals were administered by veterinarians and 39.4% of the animal producers were administered their animals by themselves. A larger proportion (53%) than our finding, the livestock farmers in African countries were administered antimicrobials by themselves [18].

In the present study, the majority (56.7%) of the farmers had the trend of using AMU guidelines/regulations. Higher than the current finding, 70% of the livestock farmers in Ethiopia [12], 93% of animal producers in Vietnam [16], and 80.3% of the dairy producers in Swiss [25] had the practice of using treatment protocol or AMU guidelines. On the contrary, 66.7% of the farmers in different regions of Thailand [14] were using antimicrobials for viral treatment without considering the recommended use of antimicrobials. As presented by the American veterinary medical association [1], judicious AMU in animal production has a crucial effect on the reduction of AMR formation and transmission. Not only over and irrational AMU in animal productions, but also using poor quality drugs in animal production is found to be an aggravating factor for AMR formation [30].

### Association of demographic data with the KAP of animal producers

The educational status of the animal producers in this study was significantly associated (p<0.05) with their knowledge, attitude, and practice towards AMU and AMR. The present finding is comparable with the studies conducted in Bingol, Turkey [13], Shandong province, China [31], and in different regions of Vietnam [16]. Unlike the current study, the study in Turkey showed that the residence of the livestock framers was associated (p<0.05) with their knowledge of AMU and AMR [13].

The dummy variables of educational level (primary, secondary, and tertiary) are negatively correlated with the knowledge of animal producers (OR = 0.18, 0.26, and 0.29 respectively). In the opposite to this finding, the study done in Vietnam [16] showed that the educational level of the farmers was positively correlated with the knowledge of farmers (OR = 2.37). Alike with the knowledge of the animal producers, their educational status was negatively correlated with their attitude towards AMU and AMR in animal production. This finding is comparable with the finding in China [31]. Comparable with the present study, the study in central Ethiopia [15] revealed that the AMU and AMR practice of the livestock producers were negatively correlated with their educational level.

In the present study, the sex of animal producers is significantly associated (p<0.05) with their knowledge and practice towards AMU and AMR. The sex of the animal producers in the current study was positively correlated with both farmer’s knowledge (OR = 3.49, 95%Cl = 2.14–5.68) and practice (OR = 2.89, 95%Cl = 1.84–4.53). The reason why male animal producers were more knowledgeable towards AMU and AMR than females might be due to the exposure of males to meetings, training, and media in the study area. Participation of females in meetings, training, and others is not culturally common in the study area. Likewise, the study conducted in Shandong province, China [31] showed that sex is associated with the knowledge and practice of AMU and AMR (males had known more than females and improperly practice than females).

## Conclusion and recommendations

From this study, the mitigation of AMR formation and effective governance of AMU along the animal origin food chain requires a thorough understanding of the animal producers’ knowledge, attitude, and practice towards AMU and AMR in animal production, using a One Health approach. The animal producers in the Oromia zone had unsatisfactory knowledge of AMU and AMR. Alike their knowledge their attitude and practice regarding AMU and AMR were majorly negative and improper respectively. Overall, the practical recommendations based on the findings from the study include:

1. Continuous awareness creation should be done to animal producers and the general community.
2. Implementable and inclusive AMU policy formulation and enforcement are recommended.
3. Integrated AMU governance among all individuals, sectors, and actors regardless of their geographic locations and economic status should be applied.
4. All GOs and NGOs should have a special focus to control imprudent AMU and AMR in animal production.

## Study limitations

The study might be liable to social desirability. In addition, the nature of the study design (cross-sectional) can influence the cause and effect relationship of the predictor variables and the dependent binary variables (knowledge, attitude, and practice) of the animal producers.

## Supporting information

S1 File The full data set that used to analyze the KAP of animal producers regarding AMU and AMR in animal production. (XLSX)

S2 File Questionnaire that was used during data collection. (PDF)
