# Supplementary material for: Knowledge, attitude and practice of animal producers towards antimicrobial use and antimicrobial resistance in Oromia zone, north eastern Ethiopia
Source: PLoS One. 2021 May 12;16(5):e0251596. doi: 10.1371/journal.pone.0251596 (PMC8115805; doi:10.1371/journal.pone.0251596)
Supplement: S2 File — (PDF) [file pone.0251596.s002.pdf]

Wollo University

School of Veterinary Medicine

Research Questioner for Assessing Community Knowledge, Attitude and Practice (KAP) Towards Antimicrobial use and Antimicrobial Resistance at Oromia Zone, North Eastern Ethiopia

This questioner survey is collected for research purpose and the data collected from the respondents and their confidentiality will be guaranteed by Wollo university research ethics guideline. The privacy regarding the knowledge, attitude and practice of AMU and AMR will be managed secretly and will going to use for research purpose only. Dear respondents, it is necessary to forward our greatest gratitude from the beginning in the name of research team, for your valuable contribution in this questioner survey.

Questionnaire Serial Number: ___________ Interviewer: _____________________________

Section I: Respondents' socio demographic information

1. ID of the respondent: _________
2. Age
   A. 18-30 B. 31-40 C. >40
3. Sex
   A. Male B. Female
4. Educational level
   A. Illiterate C. Secondary school
   B. Primary school D. Tertiary education
5. Residence:
   A. Urban B. Semi Urban C. Rural
6. District
   A. Bati B. Dewa Chefa C. Jule Timuga D. Kemissie
7. Animal type reared
   A. Cattle C. Goat E. All animal types
   B. Sheep D. Poultry

Section II: Knowledge of animal producers towards AMU and AMR in animal production.

1. Do you know or heard of about AMU and AMR?
   A. Yes B. No
2. Can zoonotic diseases causing agents develop AMR in animals?

A. Yes B. No C. I don’t know

3. Do you know using animal origin food products before the end of withdrawal period can promote AMR development in human?

   A. Yes B. No C. I don’t know

4. Can the use of antimicrobials in animal production boosts the rate of AMR development?

   A. Yes B. No C. I don’t know

5. Can you reduce AMR development by avoiding over use of antimicrobials in animal production?

   A. Yes B. No C. I don’t know

6. Can your imprudent use of antimicrobials affect the health of others in the form of AMR?

   A. Yes B. No C. I don’t know

Section III: Attitude of animal producers towards AMU and AMR in animal production

Please tick (√) your level of agreement about the questions in the following table. The meaning of the numbers in the first row is defined below.

1= strongly agree 3= neutral 5= strongly disagree

2= agree 4= disagree 6= I don’t know

	Questions	1	2	3	4	5	6
1.	Is professional advice before using antimicrobials recommended?						
2.	Can imprudent AMU result irreversible loss of drug effectiveness?						
3.	Can using antimicrobial alternatives like biosecurity, good hygienic practice and vaccination can reduce AMR development?						
4.	Do you think using of antimicrobials for the purpose of animal production is abusing antimicrobials?						
5.	Can AMU regulations will be a solution for the irrational use of antimicrobials in animal production?						
6.	Can public awareness creation reduce the development of AMR?						

Part IV: Practice of animal producers towards AMU and AMR in animal production

1. What did you do when your animals got sick?

   A. Self-treat C. Consult veterinarian

   B. Take to vet clinic D. Nothing

2. Who administer antimicrobials for your animals?

    A. Self-administration
    B. Veterinarian
    C. Local traditional healer

3. Did you refer guidelines while you administer antimicrobials for your animals?

    A. No
    B. Yes

4. Did you get prescription from veterinarians before you buy drugs?

    A. No
    B. Yes

5. For what purpose did you use antimicrobials most?

    A. Treatment
    B. Control (Metaphylaxis)
    C. Prevention (prophylaxis)
    D. Increase production

6. From where did you get antimicrobials for your animals?

    A. Local dispensers
    B. Veterinary clinic
    C. Veterinary pharmacy
